# Type of Multimorbidity and Complementary and Alternative Medicine Use among Adults

**DOI:** 10.1155/2015/362582

**Published:** 2015-01-08

**Authors:** Monira Alwhaibi, Rituparna Bhattacharya, Usha Sambamoorthi

**Affiliations:** Department of Pharmaceutical Systems and Policy, School of Pharmacy, West Virginia University, P.O. Box 9510, Morgantown, WV 26506-9510, USA

## Abstract

*Objective*. To examine the association between type of multimorbidity and CAM use among adults with multimorbidity. *Methods*. The current study used a cross-sectional design with retrospective data from 2012 National Health Interview Survey. Multimorbidity was classified into two groups: (1) adults with coexisting physical and mental illnesses and (2) adults with two or more chronic physical illnesses only. CAM use was measured using a set of 18 variables. Logistic regression and multinomial logistic regressions were used to assess the association between the type of multimorbidity and ever used CAM, CAM use in the past 12 months, and type of CAM. *Results*. Overall, 31.2% of adults with coexisting physical and mental illnesses and 20.1% of adults with only physical illnesses used CAM in the past 12 months. Adults with coexisting physical and mental illnesses were more likely to ever use CAM (AOR = 1.68, 95% CI = 1.49, 1.90), use CAM in the past 12 months (AOR = 1.32, 95% CI = 1.15, 1.52), and use mind-body therapies in the past 12 months (AOR = 1.36, 95% CI = 1.16, 1.59) compared to adults with only physical illnesses. *Conclusion*. Multimorbidity of chronic physical and mental illnesses was associated with higher CAM use.

## 1. Introduction 

The use of complementary and alternative medicine (CAM) is highly prevalent among adults with chronic conditions [1–3]. In 2002, an estimated 42% to 60% of adults with the most common chronic physical conditions used CAM [1]. Adults with multimorbidity, defined as the presence of two or more chronic conditions, had a higher percentage of CAM use as compared to adults with single or no chronic physical conditions (55% versus 44%) [1, 3–5]. Among adults with diabetes, those with two or more chronic conditions had a higher percentage (35%) of CAM use compared to adults with single or no chronic conditions (28%); however, this study did not report type of chronic conditions (physical versus mental) [3]. Adults with chronic physical conditions such as cancer and arthritis may use CAM to relieve illness-related symptoms, reduce stress, reduce pain intensity, enhance well-being, and improve the level of activity [6–8].

CAM use is also highly prevalent among adults with mental illnesses [9]. Separate body of literature has documented that adults with depression, anxiety, bipolar spectrum disorders, and schizophrenia used CAM to treat their illness [10, 11]. Adults with mental illnesses used CAM because they perceived that CAM can improve physical, emotional, social, and cognitive functioning [12]. In 2002, a cross-sectional study using the National Health Interview Survey (NHIS) found that an estimated 81.7% of older adults with anxiety or depression used CAM in the past 12 months compared to 64.6% of older adults without these mental illnesses [11]. Data from a national household telephone survey found a higher percentage of CAM use among adults with panic disorder (32.0%) and major depression (22.4%) compared to adults without these disorders (13.7%) [13].

A special case of multimorbidity occurs when chronic physical illnesses and mental illnesses cooccur [14]. Limited research studies in this area have suggested that adults with chronic physical illnesses and mental illnesses were more likely to use CAM compared to those with only physical illnesses or only mental illnesses. The Coordinated Anxiety Learning and Management (CALM) trial in adults with generalized anxiety disorder (GAD) found that adults with GAD were more likely to use CAM (adjusted odds ratio (AOR) = 1.96, 95% confidence intervals (CI): 1.26–3.02) if they have two or more chronic physical conditions compared to adults without any chronic physical conditions [15]. Among cancer patients, those with depression or anxiety were more likely to use CAM compared to those without depression and anxiety [8, 16, 17]. As the coexistence of chronic physical conditions and mental illnesses has been found to be associated with work absenteeism, functional disability, preventable hospitalization, and poor quality of life [14, 18–25], it is plausible that adults with chronic physical and mental illnesses are more likely to use CAM compared to those with multiple chronic physical illnesses only.

While the above-mentioned studies have highlighted higher CAM use among adults with chronic physical illnesses and mental illnesses, they were limited to specific physical illnesses (such as those with cancer) [8, 16, 17] or mental illnesses (such as those with only GAD) [15]. In addition, some of these studies were restricted to specific geographic regions [8, 15] and to those who were seeking care in primary care settings [15]. Therefore, the primary objective of this study was to examine the association between type of multimorbidity and CAM use among adults with highly prevalent chronic physical illnesses, using data from a nationally representative household survey. An understanding of the relationship between type of multimorbidity and CAM use is important because of the growing prevalence of multimorbidity in the US in all age groups [26]. It has been projected that eighty-one million Americans will be living with multiple chronic conditions by 2020 (25% of Americans) [27]. Based on results observed in the limited previous research, it is hypothesized that adults with coexisting chronic physical and mental illnesses will be more likely to use CAM as compared to those with multiple chronic physical illnesses only.

## 2. Methods

### 2.1. Study Design

A retrospective cross-sectional study of adults with at least two chronic physical illnesses or a combination of any chronic physical condition and any mental illness was conducted.

### 2.2. Data Source

The current study used 2012 NHIS. The NHIS is an annual survey of households in the US. The survey is available in two types of files, the core and the supplements [28]. The core consists of four main sections: the Household Composition, the Family Core, the Sample Child Core, and the Sample Adult Core. Adult members of the household (≥18 years old) are invited to complete the Family Core component, while a randomly selected adult family member is selected to complete the Sample Adult Core. This study used data from participant responses to questions in the Family Core, the Sample Adult Core, and the Adult Complementary and Alternative Medicine Supplement. The Family Core provides information about sociodemographic characteristics, health status, health insurance, access to care, and utilization of health care services. Chronic physical and mental illnesses were captured from the Sample Adult Core file; for example, sample adults were asked whether they have ever been told by a doctor or another health professional that they had asthma. Adult Complementary and Alternative Medicine (ALT) Supplements file was used to obtain information about CAM use. In the ALT supplements, file respondents were asked whether they ever used CAM, and if so, whether they used CAM in the past 12 months. For each type of CAM, respondents were asked whether they ever used CAM, and if so, whether they ever used the type of CAM, and if they responded yes, whether they ever used the type of CAM in the past 12 months.

### 2.3. Study Sample

The study sample is comprised of adults, aged > 21 years, who were part of Sample Adult Core and responded to CAM supplementary file. The study sample was further restricted to adults who reported having at least one chronic physical condition and at least one mental illness or reported having two or more chronic physical illnesses without any mental illnesses. Individuals with missing data on CAM use variables were excluded from the sample. The final study sample consisted of 13,246 adults with multimorbidity.

### 2.4. Measures

#### 2.4.1. Dependent Variables

*Ever Used CAM*. In this study, CAM use was derived from a set of 18 variables (homeopathy, acupuncture, naturopathy, Ayurveda, chiropractic or osteopathic manipulation, massage, Feldenkrais, alexander technique, trager psychophysical integration, craniosacral therapy, pilates, biofeedback, hypnosis, yoga, tai chi, qi gong, energy healing therapy, and chelation therapy). Adults who reported using any of the above-mentioned types of CAM were considered to have “ever used CAM.” Adults who used none of the 18 types of CAM were considered to have “never used CAM.” Adults with missing data on all the 18 types were not eligible to be included in the sample.

*CAM Use in the Past 12 Months*. Among individuals who ever used CAM, CAM use in the past 12 months was defined. Adults who reported using at least one of the 18 CAM types in the past 12 months were considered to have “used CAM in the past 12 months.” Adults who used none of the 18 types of CAM in the past 12 months were considered as “not used CAM in the past 12 months.” 

*Types of CAM*. Among adults who ever used CAM, three types of CAM use were further studied. These were (1) alternative medical systems which included homeopathy, acupuncture, naturopathy, and Ayurveda, (2) manipulative and body-based therapies which included chiropractic or osteopathic manipulation, massage, Feldenkrais, alexander technique, trager psychophysical integration, craniosacral therapy, and pilates, and (3) mind-body therapies which included biofeedback, hypnosis, yoga, tai chi, and qi gong.

Adults who used any of the four types of alternative medical systems were considered to have “ever used alternative medical systems.” Adults who used none of the four types of alternative medical systems were considered to have “never used alternative medical systems.” Among adults who used alternative medical systems, its use in the past 12 months was defined for each type of alternative medical systems. Adults who reported using any of the alternative medical systems types were considered to have “used alternative medical systems in the past 12 months.” Adults who reported using none of the alternative medical systems types were considered as “not used alternative medical systems in the past 12 months.” This variable was grouped into four categories: (1) used alternative medical systems in the past 12 months; (2) not used alternative medical systems in the past 12 months; (3) never used alternative medical systems; and (4) never used CAM. Similarly, manipulative and body-based therapies and mind-body therapies were grouped into four categories.

#### 2.4.2. Key Independent Variable: Type of Multimorbidity

Type of multimorbidity was classified into two groups: (1) presence of at least one chronic physical condition with at least one mental illness (coexisting physical and mental illnesses) and (2) presence of two or more chronic physical conditions only (physical illnesses only). Chronic physical illnesses included asthma, arthritis, cancer, COPD, diabetes, heart diseases (angina pectoris, coronary heart disease, heart attack, stroke, and other heart conditions), hyperlipidemia, or hypertension. These illnesses were selected because of high clinical and economic burden in which mental illnesses are highly prevalent [29, 30]. Mental illnesses included bipolar disorder, depression, or other mental health disorders.

#### 2.4.3. Other Independent Variables

Based on the modified version of Anderson Healthcare Utilization Model [31–34], variables that were used to examine the factors associated with CAM use included the following. (1) Predisposing factors included age groups in years (22–39, 40–49, 50–64, and more than 65), gender, race/ethnicity (white, African American, Latino, and others), education level (less than high school, high school, and greater than high school), and marital status (married, widowed/divorced/separated, and never married). (2) Enabling factors included health insurance coverage (insured and uninsured) and poverty status. Poverty status variable was defined as poor (less than 100% federal poverty line), near poor (100% to less than 125%), middle income (200% to less than 400%), and high income (greater than or equal to 400%). (3) Need factors consisted of perceived general health status (excellent, very good, good, fair, and poor), functional limitations, and the type of multimorbidity. (4) Personal health practices included body mass index (BMI) categories (underweight (0–18.5 kg/m^2^); normal (18.5–25.0 kg/m^2^); overweight (25.0–30.0 kg/m^2^); and obese (30.0–40.0 kg/m^2^)), smoking status (nonsmoker, past smoker, and current smoker), alcohol use (lifetime abstainer, former drinker, and current drinker), and physical activity (daily, weekly, monthly/yearly, and unable to exercise). (5) External environmental factor was region of residence (northwest, midwest, south, and west region).

### 2.5. Statistical Analysis

Chi-square tests were used to examine significant differences in ever used CAM, CAM use in the past 12 months, type of CAM, and the type of multimorbidity (coexisting physical and mental illnesses and physical illnesses only). Logistic regression was used to assess the relationship between the type of multimorbidity and ever used CAM after adjusting for predisposing, enabling, need, external environment factors, and personal health practices. Multinomial logistic regressions were used to assess the association between the type of multimorbidity and CAM use in the past 12 months; type of multimorbidity; and the type of CAM used (alternative medical systems, manipulative and body-based therapies, and mind-body therapies) after adjusting for predisposing, enabling, need factors, external environment factors, and personal health practices. All analyses controlled for the complex survey design of NHIS and were conducted using survey procedure with Statistical Analysis System Software (SAS 9.4 Institute Inc., Cary, NC, USA).

## 3. Results

### 3.1. Description of the Study Sample

Table 1 displays the characteristics of the study sample (*N* = 13,246). The majority of the samples were women (54.4%) and white (75.1%). Nearly one-third (33%) had high income measured as 400% above the federal poverty line, 56.2% had above high school education, and 9.9% were uninsured. Only 34.6% perceived their general health to be excellent, and 62.0% had functional limitations.

Table 1 provides a description of CAM use by subject characteristics. In our sample, 48.2% reported ever using CAM and 23.6% reported CAM use in the past 12 months. Approximately 5% used alternative medical systems, 18.0% used manipulative and body-based therapies, and 8.0% used mind-body therapies. There were significant differences in predisposing, enabling, need, external environmental factors, and personal health practices between adults who ever used CAM. Ever used CAM was reported by a significantly higher percentage of women (51.5%), whites (52.4%), young adults in the age group 22–29 years (52.4%), adults with high income (58.0%), those with excellent perceived general health status (55.5%), those with functional limitations (50.0%), and past smokers (51.6%) as compared to men (44.3%), African Americans (31.1%), older adults (≥65 years) (42.7%), adults with low income (34.0%), those with poor perceived general health status (38.7%), those with no functional limitations (45.4%), and current smokers (45.1%).

### 3.2. Type of Multimorbidity and CAM Use

A statistically significant association between the type of multimorbidity and ever used CAM, CAM used in the past 12 months, and the types of CAM used was observed (Table 2). As compared to adults with physical illnesses only, a higher percentage of adults with coexisting physical and mental illnesses reported ever using CAM (44.1% versus 57.1%), CAM use in the past 12 months (20.1% versus 31.2%), alternative medical systems (3.6% versus 6.7%), manipulative and body-based therapies (15.3% versus 24.1%), and mind-body therapies (6.1% versus 12.1%).

### 3.3. Type of Multimorbidity and CAM Use: Multivariable Logistic Regression

Adjusted odds ratios (AORs) and 95% confidence intervals (CI) of type of multimorbidity from logistic regressions on CAM use after controlling for predisposing, enabling, need, external environmental factors, and personal health practices are presented in Table 3. Adults with coexisting physical and mental illnesses were 1.68 times as likely to have ever used CAM (AOR = 1.68, 95% CI = 1.49, 1.90) compared to adults with physical illnesses only. Adults with coexisting physical and mental illnesses were 1.32 times as likely to report any CAM use in the past 12 months (AOR = 1.32, 95% CI = 1.15, 1.52) compared to adults with physical illnesses only. With regard to the types of CAM, it was observed that adults with coexisting physical and mental illnesses were 1.36 times as likely to report use of manipulative and body-based therapies in the past 12 months (AOR = 1.36, 95% CI = 1.16, 1.59) as those with physical illnesses only.

## 4. Discussion

The purpose of the present study was to examine the association between the type of multimorbidity and CAM use. We found that in our sample 48.2% of adults ever used CAM and 23.6% used CAM in the past 12 months. Among adults with multimorbidity, those with coexisting physical and mental illnesses were more likely to use CAM compared to adults with physical illnesses only. Several reasons could lead to greater CAM use among adults with coexisting physical and mental illnesses. As shown in previous studies, adults with coexisting physical and mental illnesses have higher functional disabilities, pain, and poor quality of life compared to those with physical conditions only [24, 35, 36]. For example, worldwide adults with coexisting chronic physical and mental illness were more likely to have severe disability compared to a single physical illness or a mental illness [36]. Therefore, adults with coexisting physical and mental illnesses may use CAM to improve their functional status. The Sequenced Treatment Alternatives to Relieve Depression (STAR^*^d) study, the largest prospective study of sequential series of treatment for depression, has reported that only one-third of patients get relief of their depressive symptoms with initial antidepressant treatment [37, 38]. Therefore, adults with coexisting physical and mental illnesses may be more likely to use CAM if they do not get relief from conventional therapies or if they develop side effects from the conventional therapies [39]. A national telephone survey of women with depression found that 43% of women reported that the reason for using CAM was the ineffectiveness of the conventional therapy and 45% of women reported the reason for using CAM was the side effects of the conventional therapies [39].

Among types of CAM used in the past 12 months, manipulative and body-based therapies were the most common (18%) followed by mind-body therapies (8%) and alternative medical systems (5%). We also found that as compared to adults with physical illnesses only, those with coexisting physical and mental illnesses were more likely to use manipulative and body-based therapies in the past 12 months. Insurance coverage for manipulative and body-based therapies (e.g., chiropractic care) may be one of the reasons for the higher use of manipulative and body-based therapies as compared to other types of CAM use. For example, chiropractic care is covered by 45 states in the US as an essential benefit, while other therapies such as acupuncture, type of alternative medical systems, are covered by only six states [40]. The reimbursement for chiropractic care for Medicare beneficiaries is 80% [41]. At least 75% of private payers and 50% of managed care organizations cover chiropractic care [42].

In the current study, a substantial percentage (48.2%) of the adults with multimorbidity used CAM. The safety of some types of CAM therapies has not been established and some CAM therapies lead to some side effects [43, 44]. In addition, the clinical efficacy and effectiveness of many of the CAM therapies for treating chronic conditions have not been established [45–47]. For example, a systematic review of clinical trials on the efficacy of CAM use in relieving cancer pain reported that while CAM therapies such as hypnosis, acupuncture, and imagery were promising, there is a need for rigorous trials to establish the efficacy of these therapies [45]. Another systematic review of the efficacy and effectiveness of CAM therapies for the treatment of rheumatoid arthritis has reported that there are few studies evaluating the efficacy and effectiveness of CAM therapies [46]. Of the effectiveness studies that were reported, only one study showed that tai chi, a mind-body therapy, was effective for the treatment of rheumatoid arthritis [46]. Therefore, more research is needed to establish the safety, efficacy, and effectiveness of CAM therapies. Adults with some chronic physical and mental health illnesses need to be cautious and consult with their healthcare providers before using any CAM therapy [47].

The study had many advantages. The study used nationally representative data with large sample size, included adults with multimorbidity, and evaluated the association between type of multimorbidity and CAM use after controlling for a comprehensive list of factors. Results of this study should be interpreted in the context of some limitations. Owing to the cross-sectional nature of the data it is difficult to assess the causal relationship. All measures in the study were self-reported and thus subject to recall bias. Furthermore, many variables such as the severity of the chronic illnesses, pain and attitude towards CAM that may affect CAM use were not measured.

## 5. Conclusion

Despite limitations, the current study found that, among adults with multimorbidity, those with coexisting chronic physical and mental illnesses were more likely to use CAM compared to those with two or more chronic physical illnesses. Given the increasing prevalence of coexisting chronic physical and mental illnesses among adults [27], medical providers and payers may need to consider an integrative medicine approach that includes conventional and effective CAM therapies in treating patients with multimorbidity. In addition, future research needs to evaluate the effectiveness of CAM therapies among those with multimorbidity.

## Figures and Tables

**Table 1:** Description of study sample and number and weighted percent with complementary and alternative medicine use, National Health Interview Survey 2012.

	Total sample		Ever used CAM		Sig.
	*N*	Wt. %	*N*	Wt. %	
All	**13,246**	**100.0**	**6,212**	**48.2**	
Gender					∗∗∗
Women	7,738	54.4	3,839	51.5	
Men	5,508	45.6	2,373	44.3	
Race/ethnicity					∗∗∗
White	8,941	75.1	4,710	52.4	
African American	2,125	11.3	644	31.1	
Latino	1,476	9.3	535	36.2	
Other races	704	4.3	323	45.8	
Age in years					∗∗∗
22–39 years	1,545	12.1	809	52.4	
40–49 years	1,711	14.9	865	50.5	
50–64 years	4,658	36.9	2,322	51.4	
65 and older	5,332	36.1	2,216	42.7	
Marital status					∗∗∗
Married	6,346	63.1	3,125	50.4	
Wid./Div./Sep.	5,154	26.9	2,293	44.4	
Never married	1,724	10.0	781	44.8	
Education level					∗∗∗
LT high school	2,421	15.6	695	29.0	
High school	3,709	28.1	1,491	42.0	
GT high school	7,067	56.2	4,016	56.9	
Poverty status					∗∗∗
Poor	2,239	12.4	730	34.0	
Near poor	2,565	16.4	1,066	41.5	
Middle income	3,321	26.0	1,638	48.6	
High income	3,588	33.0	2,122	58.0	
Missing	1533	12.2	656	44.6	
Insurance					
Insured	11,869	90.1	5,578	48.4	
Uninsured	1,352	9.9	625	46.7	
General health					∗∗∗
Excellent	1,409	11.6	776	55.5	
Very good	3,351	27.5	1,786	53.2	
Good	4,628	34.6	2,154	47.9	
Fair	2,801	19.0	1,095	41.0	
Poor	1,050	7.3	400	38.7	
Functional limitation					∗∗∗
Yes	8,535	62.0	4,091	50.0	
No	4,700	38.0	2,118	45.4	
Body mass index					
Underweight	180	1.2	75	43.7	
Normal weight	3,204	23.1	1,555	50.6	
Overweight	4,521	35.1	2,120	47.7	
Obese	4,957	37.5	2,275	47.5	
Missing	384	3.1	187	47.6	
Smoking status					∗∗∗
Never smoke	6,473	48.4	2,987	47.1	
Past smoker	4,178	33.0	2,098	51.6	
Current smoker	2,575	18.6	1,121	45.1	
Alcohol drinking					∗∗∗
Lifetime abstainer	2,795	18.7	952	34.4	
Former drinker	5,004	37.2	2,313	47.8	
Current drinker	5,343	43.4	2,911	54.7	
Missing	104	0.7	36	35.1	
Physical activity					∗∗∗
Daily	735	5.4	396	55.3	
Weekly	2,970	24.8	1,779	59.3	
Monthly/yearly	8,868	65.2	3,782	44.2	
Unable to do	590	4.0	218	36.2	
Missing	83	0.6	37	45.0	
Region					∗∗∗
Northeast	2,193	17.2	1,003	45.2	
Midwest	2,814	23.4	1,484	53.3	
South	5,021	38.2	1,887	40.8	
West	3,218	21.3	1,838	58.5	

*Note.* Based on 13,246 adults, age over 21 years, having at least two or more chronic physical illnesses or one or more chronic physical illnesses with mental illness. Chronic physical illnesses consisted of diabetes, heart disease, hyperlipidemia, hypertension, arthritis, cancer, and respiratory diseases. Mental illnesses consisted of depression, bipolar disorder, or other mental health disorders. Percentages may not add to 100 due to missing data in marital status, education level, insurance, general health, functional status, and smoking status. Asterisks represent significant group differences by complementary and alternative medicine use based on chi-square tests.

Wt.: weighted; CAM: complementary and alternative medicine; LT: less than; GT: greater than; Wid./Div./Sep.: widowed, divorced, and separated. ^***^ *P* < .001; ^**^.001 ≤ *P* < .01; ^*^.01 ≤ *P* < .05.

**Table:** (a) Number and weighted percent of ever used CAM (*N* = 13,246)

	Ever used CAM		Never used CAM		Sig.
	*N*	Wt. %	*N*	Wt. %	
All	6,212	48.2	7,034	51.8	
Multimorbidity					∗∗∗
PI & MI	2,439	57.1	1,931	42.9	
PI only	3,773	44.1	5,103	55.9	

**Table:** (b) Number and weighted percent of CAM uses in the past 12 months (*N* = 13,246)

	CAM use in the past 12 months		No CAM use in the past 12 months		Never used CAM		Sig.
	*N*	Wt. %	*N*	Wt. %	*N*	Wt. %	
All	3,037	23.6	3,175	24.6	7,034	51.8	
Multimorbidity							∗∗∗
PI & MI	1,301	31.2	1,138	25.9	1,931	42.9	
PI only	1,736	20.1	2,037	24.0	5,103	55.9	

**Table:** (c) Number and weighted percent of alternative medical systems use in the past 12-month (*N* = 13,215)

	Alternative medical systems use in the past 12 months		No alternative medical systems use in the past 12 months		Never used alternative medical systems		Never used CAM		Sig.
	*N*	Wt. %	*N*	Wt. %	*N*	Wt. %	*N*	Wt. %	
All	608	4.5	1,117	8.0	4,436	35.5	7,034	51.9	
Multimorbidity									∗∗∗
PI & MI	283	6.4	482	11.0	1,662	39.6	1,931	43.0	
PI only	325	3.6	628	6.5	2,801	33.9	5,103	56.0	

**Table:** (d) Number and weighted percent of manipulative and body-based therapies use in the past 12-month (*N* = 13,223)

	Manipulative and body-based therapies in the past 12 months		No manipulative and body-based therapies in the past 12 months		Never used manipulative and body-based therapies		Never used CAM		Sig.
	*N*	Wt. %	*N*	Wt. %	*N*	Wt. %	*N*	Wt. %	
All	2,295	18.1	3,044	23.7	859	6.4	7,034	51.8	
Multimorbidity									∗∗∗
PI & MI	993	24.1	1,109	25.4	327	7.5	1,931	43.0	
PI only	1,302	15.3	1,934	23.0	522	5.7	5,103	56.0	

**Table:** (e) Number and weighted percent of mind-body therapies use in the past 12-month (*N* = 13,212)

	Used mind-body therapies in the past 12 months		No mind-body therapies use in the past 12 months		Never used mind-body therapies		Never used CAM		Sig.
	*N*	Wt. %	*N*	Wt. %	*N*	Wt. %	*N*	Wt. %	
All	1,033	8.0	1,003	7.6	4,142	32.5	7,034	51.9	
Multimorbidity									∗∗∗
PI & MI	500	12.1	463	10.4	1,464	34.5	1,931	43.0	
PI only	533	6.1	540	6.3	2,678	31.6	5,103	56.0	

*Note.* Based on 13,246 adults, age over 21 years, having at least two or more chronic physical illnesses or one or more chronic physical illnesses with mental illness. Chronic physical illnesses consisted of diabetes, heart disease, hyperlipidemia, hypertension, arthritis, cancer, respiratory disease (chronic obstructive pulmonary disease (COPD)), and asthma. Mental illnesses consisted of depression, bipolar disorder, or other mental health disorders. Number across complementary and alternative medicine groups may not add to 12,246 due to missing data in type of complementary alternative medicine. Asterisks represent significant group differences by complementary and alternative medicine use based on chi-square tests.

Wt.: weighted; CAM: complementary and alternative medicine; PI & MI: chronic physical and mental illnesses; PI: chronic physical illnesses.

^***^ *P* < .001; ^**^.001 ≤ *P* < .01; ^*^.01 ≤ *P* < .05.

**Table:** (a) Logistic regression on any CAM use (reference: never used CAM)

	AOR	95% CI	Sig.
Multimorbidity			
PI only (Ref.)			
PI & MI	1.68	[1.49, 1.90]	∗∗∗

**Table:** (b) Multinomial logistic regression on CAM use in the past 12 months (reference: no CAM use in the past 12 months)

	Past 12 months		Sig.	Never used CAM		Sig.
	AOR	95% CI		AOR	95% CI	
Multimorbidity						
PI only (Ref.)						
PI & MI	1.32	[1.15, 1.52]	∗∗∗	0.68	[0.59, 0.78]	∗∗∗

**Table:** (c) Multinomial logistic regression on alternative medical systems use in the past 12 months (reference: no alternative medical systems use in the past 12 months)

	Used alternative medical systems in the past 12 months		Sig.	Never used alternative medical systems		Sig.	Never used CAM		Sig.
	AOR	95% CI		AOR	95% CI		AOR	95% CI	
Multimorbidity									
PI only (Ref.)									
PI & MI	0.95	[0.71, 1.26]		0.71	[0.59, 0.85]	∗∗∗	0.46	[0.37, 0.56]	∗∗∗

**Table:** (d) Multinomial logistic regression on manipulative and body-based therapies use in the past 12 months (reference: no manipulative and body-based therapies use in the past 12 months)

	Used manipulative and body-based therapies in the past 12 months		Sig.	Never used manipulative and body-based therapies		Sig.	Never used CAM		Sig.
	AOR	95% CI		AOR	95% CI		AOR	95% CI	
Multimorbidity									
PI only (Ref.)									
PI & MI	1.36	[1.16, 1.59]	∗∗∗	1.10	[0.88, 1.37]		0.67	[0.58, 0.77]	∗∗∗

**Table:** (e) Multinomial logistic regression on mind-body therapies use in the past 12 months (reference: no mind-body therapies use in the past 12 months)

	Used mind-body therapies in the past 12 months		Sig.	Never used mind-body therapies		Sig.	Never used CAM		Sig.
	AOR	95% CI		AOR	95% CI		AOR	95% CI	
Multimorbidity									
PI only (Ref.)									
PI & MI	1.08	[0.86, 1.36]		0.71	[0.58, 0.86]	∗∗∗	0.47	[0.38, 0.58]	∗∗∗

*Note.* Based on 13,246 adults, age over 21 years, having at least two or more chronic physical illnesses or one or more chronic physical illnesses with mental illness. Chronic physical illnesses consisted of diabetes, heart disease, hyperlipidemia, hypertension, arthritis, cancer, respiratory disease (chronic obstructive pulmonary disease (COPD)), and asthma. Mental illnesses consisted of depression, bipolar disorder, or other mental health disorders. Asterisks represent significant group differences compared to the reference group based on logistic regression and multinomial logistic regressions on any CAM use, CAM use in the past 12 months, and type of CAM after controlling for predisposing, enabling, need factors, external environment factors, and personal health practices.

AOR: adjusted odds ratios; CI: confidence interval; CAM: complementary and alternative medicine; Ref.: reference group; PI & MI: chronic physical and mental illnesses; PI: chronic physical illnesses.

^***^ *P* < .001; ^**^.001 ≤ *P* < .01; ^*^.01 ≤ *P* < .05.
